# Supplementary material for: Single-Cell Analysis with Silver-Coated Pipette by Combined SERS and SICM
Source: Cells. 2023 Oct 25;12(21):2521. doi: 10.3390/cells12212521 (PMC10650894; doi:10.3390/cells12212521)
Supplement: Supplementary file 1 [file cells-12-02521-s001.zip › cells-2613337-supplementary.pdf]

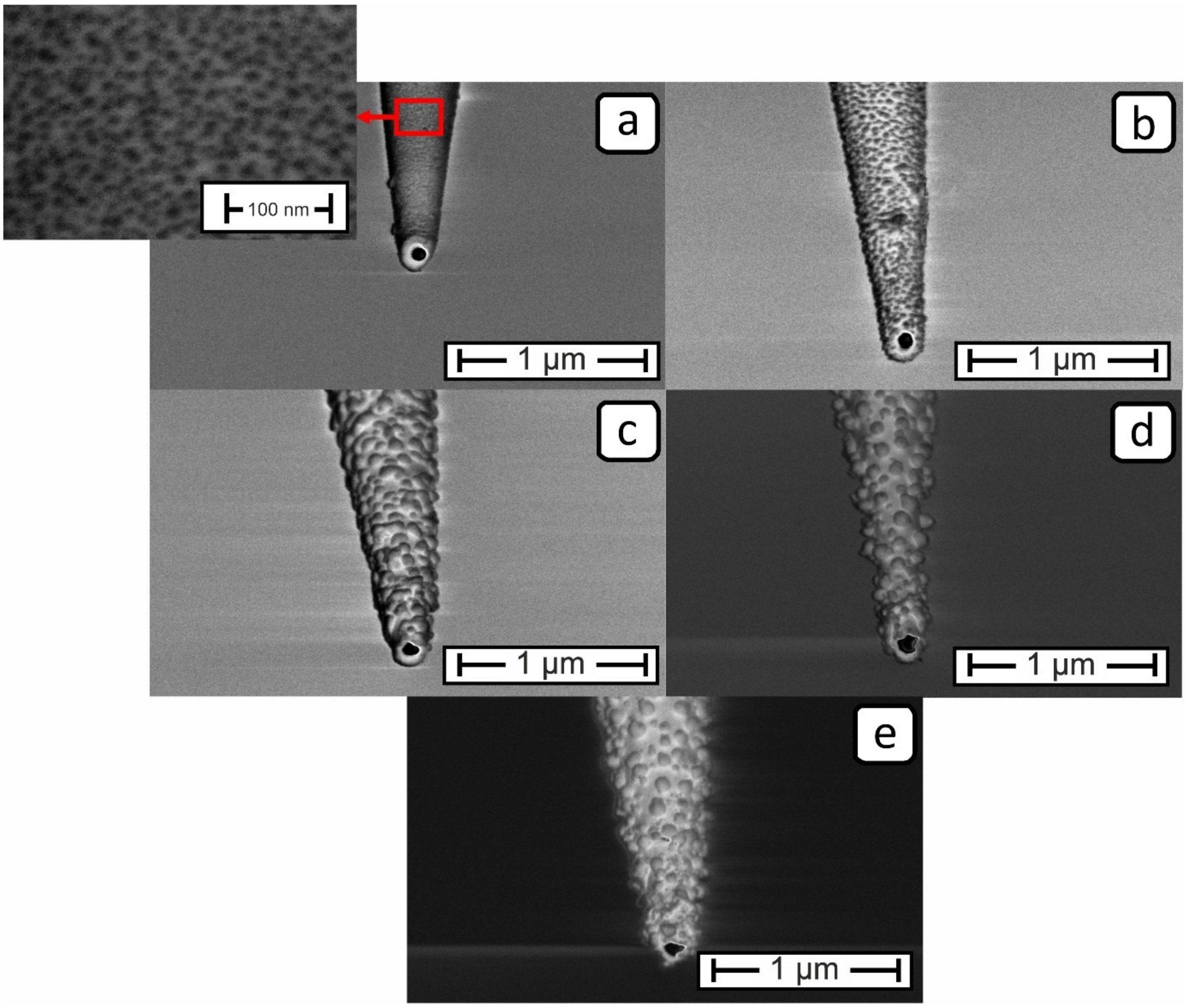

Figure S1. SEM-images of modified pipettes (a) 3 mg Ag evaporation at speed of 14 rps; (b) 15 mg Ag evaporation at speed of 14 rps; (c) 30 mg Ag evaporation at speed of 14 rps; (d) 30 mg Ag evaporation at speed of 4 rps; (e) 30 mg Ag evaporation at speed of 28 rps

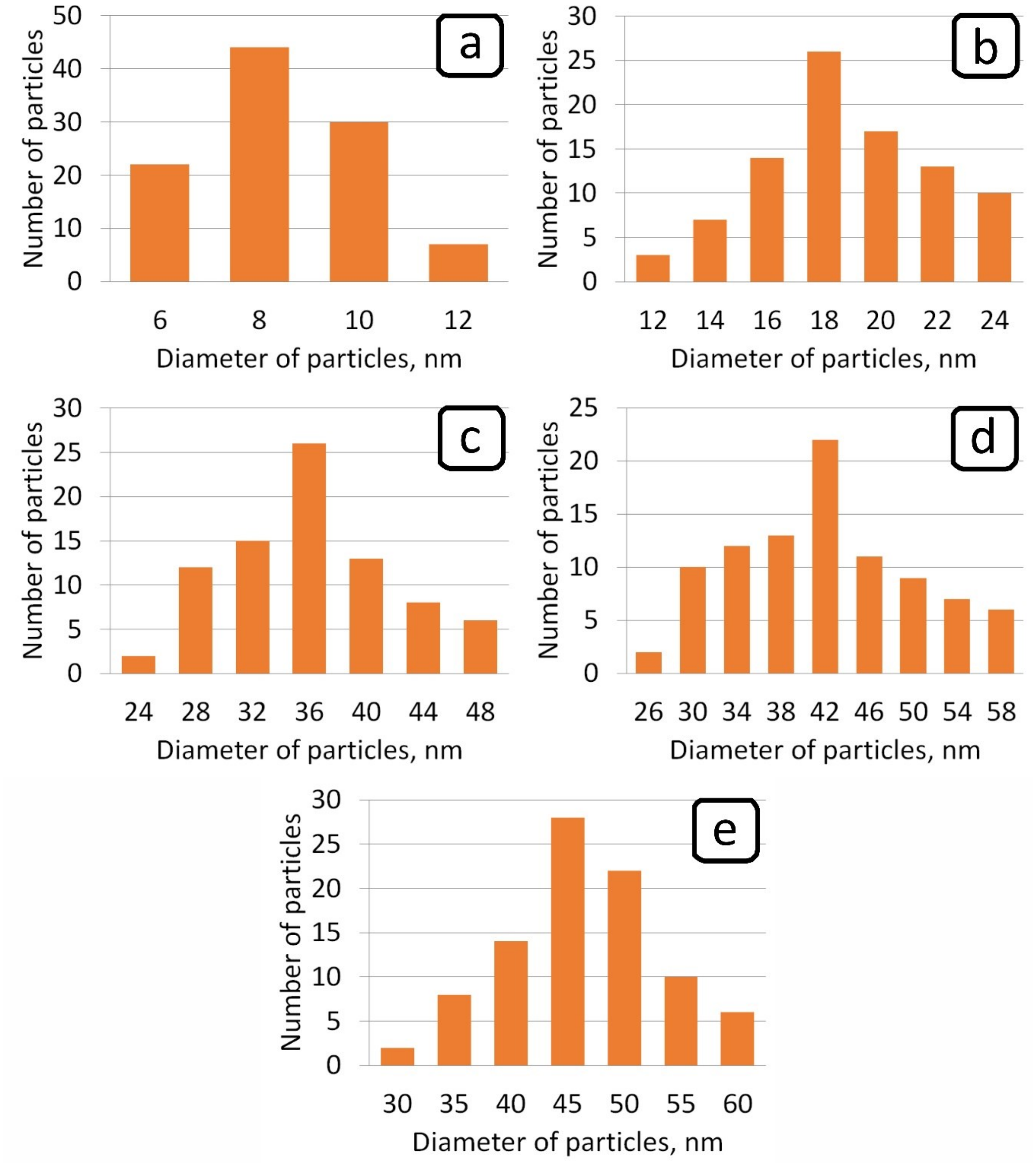

Figure S2. Histogram of nanoparticle diameter distribution (a) 3 mg Ag evaporation at speed of 14 rps; (b) 15 mg Ag evaporation at speed of 14 rps; (c) 30 mg Ag evaporation at speed of 14 rps; (d) 30 mg Ag evaporation at speed of 4 rps; (e) 30 mg Ag evaporation at speed of 28 rps

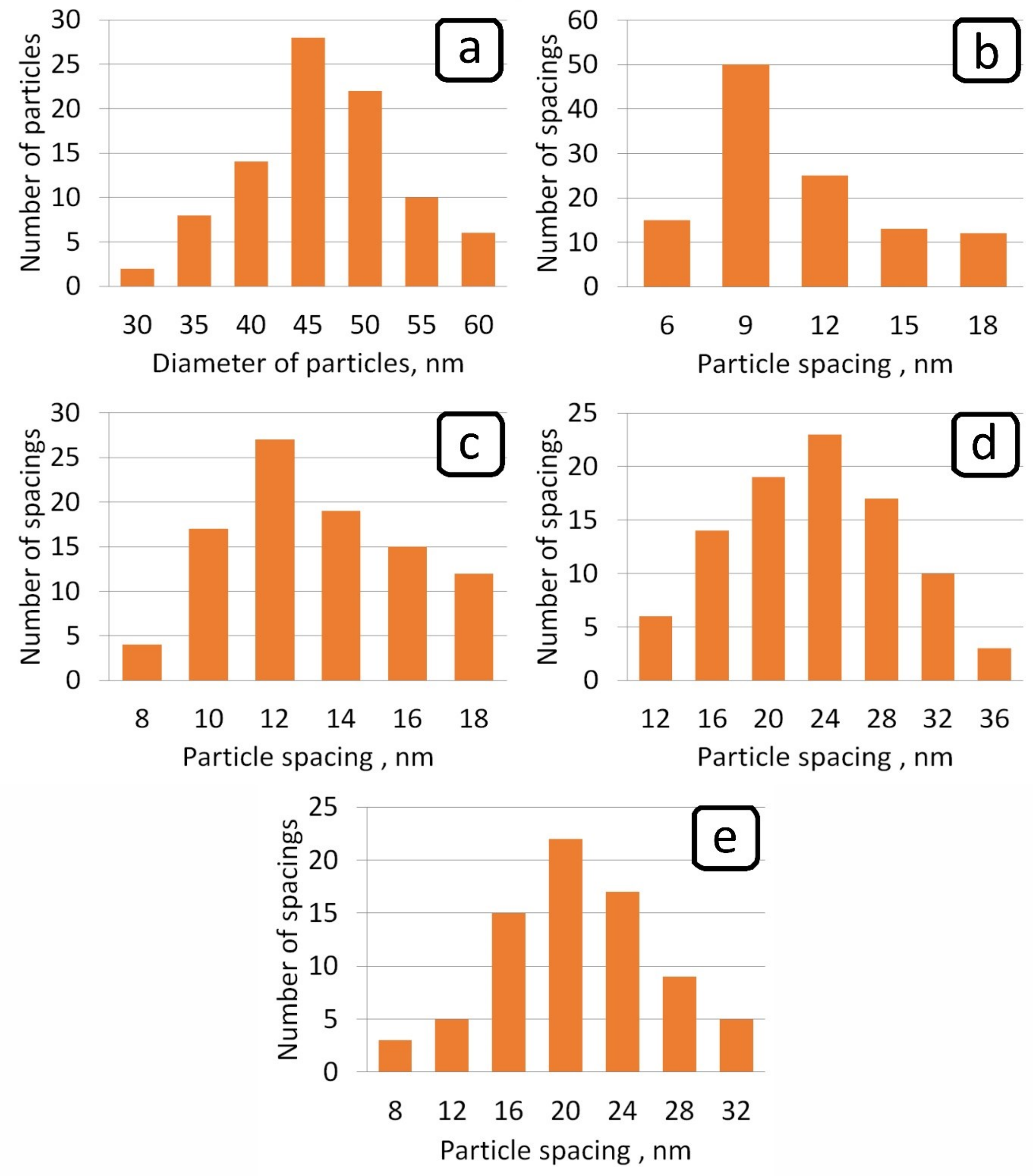

Figure S3. Histogram of nanoparticle spacing distribution (a) 3 mg Ag evaporation at speed of 14 rps; (b) 15 mg Ag evaporation at speed of 14 rps; (c) 30 mg Ag evaporation at speed of 14 rps; (d) 30 mg Ag evaporation at speed of 4 rps; (e) 30 mg Ag evaporation at speed of 28 rps

Table S1 shows a comparison of the enhancement factors of the SERS-structure obtained in this work and the closest SERS-structures presented in the works of other researchers. Based on the obtained values, it can be seen that the EF of the structure from this work is comparable to the close analogs.

Table S1. SERS-structures similar to the modified nanopipette and their enhancement factors

Structure	Enhancement factor
Pipette coated with an array of silver nanoparticles (This work)	7×10^5
Glass pipette with a layer of gold[66]	$1,5\times10^4$
Glass pipette with gold nanoporous membrane at the end[67]	2×10^6
Array of gold-silver nanoparticles on a glass substrate[68]	10^5
Thin film of silver on a nanostructured surface of a glass substrate[69]	3×10^5
Glass fiber coated with an array of silver nanoparticles[70]	$2,5\times10^4$
Glass cylindrical threads coated with an array of silver nanoparticles[71]	10^5

Table S2. Raman spectra of HEK 293 cell

Peak position, cm^{-1}	Assignment	Reference
1017	phenylalanine	[60, 62]
1174	tyrazine, guanine, phenylalanine	[60-62]
1207	tyrazine, guanine, phenylalanine	[60-62]
1285	amide III group	[60, 62]
1300	CH2 bending mode in proteins and lipids	[62, 63]
1328	DNA nucleic acid fragments	[62]
1381	CH3 bending	[60]
1410	asparagic and glutamic acids	[60]
1436	CH2 bending in lipids	[62]
1480	amide II group	[62]

1524	carotenoids not characteristic of normal tissues	[62]
1550	tryptophan	[62]
1578	guanine and adenine	[60, 62]
1600	amide I group	[62]
1610	amide I group	[62]

Table S3. Raman spectra of Hoechst 33342

Peak position, cm^{-1}	Motion	Reference
980	benzene ring bending	[64]
1358	C-C	[64]
1456	C-C stretching in benzene ring	[64]
1610	C-C stretching in phenol	[64]
